# Tailoring Functional Micromotors for Sensing

**DOI:** 10.34133/research.0044

**Published:** 2023-01-30

**Authors:** Lijun Cai, Dongyu Xu, Zeyou Zhang, Ning Li, Yuanjin Zhao

**Affiliations:** ^1^ Department of Rheumatology and Immunology, Nanjing Drum Tower Hospital, School of Biological Science and Medical Engineering, Southeast University, Nanjing 210096, China.; ^2^ Oujiang Laboratory (Zhejiang Lab for Regenerative Medicine, Vision and Brain Health), Wenzhou Institute,University of Chinese Academy of Sciences, Wenzhou, Zhejiang 325001, China.

## Abstract

Micromotors are identified as a promising candidate in the field of sensing benefiting from their capacity of autonomous movement. Here, a review on the development of tailoring micromotors for sensing is presented, covering from their propulsion mechanisms and sensing strategies to applications. First, we concisely summarize the propulsion mechanism of micromotors involving fuel-based propulsion and fuel-free propulsion introducing their principles. Then, emphasis is laid to the sensing stratagems of the micromotors including speed-based sensing strategy, fluorescence-based sensing strategy, and other strategies. We listed typical examples of different sensing stratagems. After that, we introduce the applications of micromotors in sensing fields including environmental science, food safety, and biomedical fields. Finally, we discuss the challenges and prospects of the micromotors tailored for sensing. We believe that this comprehensive review can help readers to catch the research frontiers in the field of sensing and thus to burst out new ideas.

## Introduction

Micromotors have come to the forefront as a powerful tool in scientific areas due to their unique capacity of converting diverse energy into efficient autonomous movement [1–4]. With tremendous scientific attention, miscellaneous micromotors in different morphologies have been devised for self-propelling under diverse propulsion mechanisms [5–9]. These emerging micromotors have sprung up to various fields including drug delivery, analytical sensing, and tissue regeneration [10–14]. Especially, micromotors are identified as a promising candidate in the field of sensing due to their capacity of autonomous movement in the medium [15–18]. To be specific, the micromotors can serve as ideal sensors in samples of ultrasmall volume benefiting from their tiny size [19–21]. Besides, the dynamic movement can realize efficient mixing of the sample solution, thus conducing to the reduction of analysis time and negligible sample treatment [22]. Attracted by these features, intensive investigation has been focused on tailoring micromotors for sensing [23–25]. Although much progress has been made, the fascinating design and applications of the micromotors in sensing field are rarely reviewed.

**Fig. 1.**
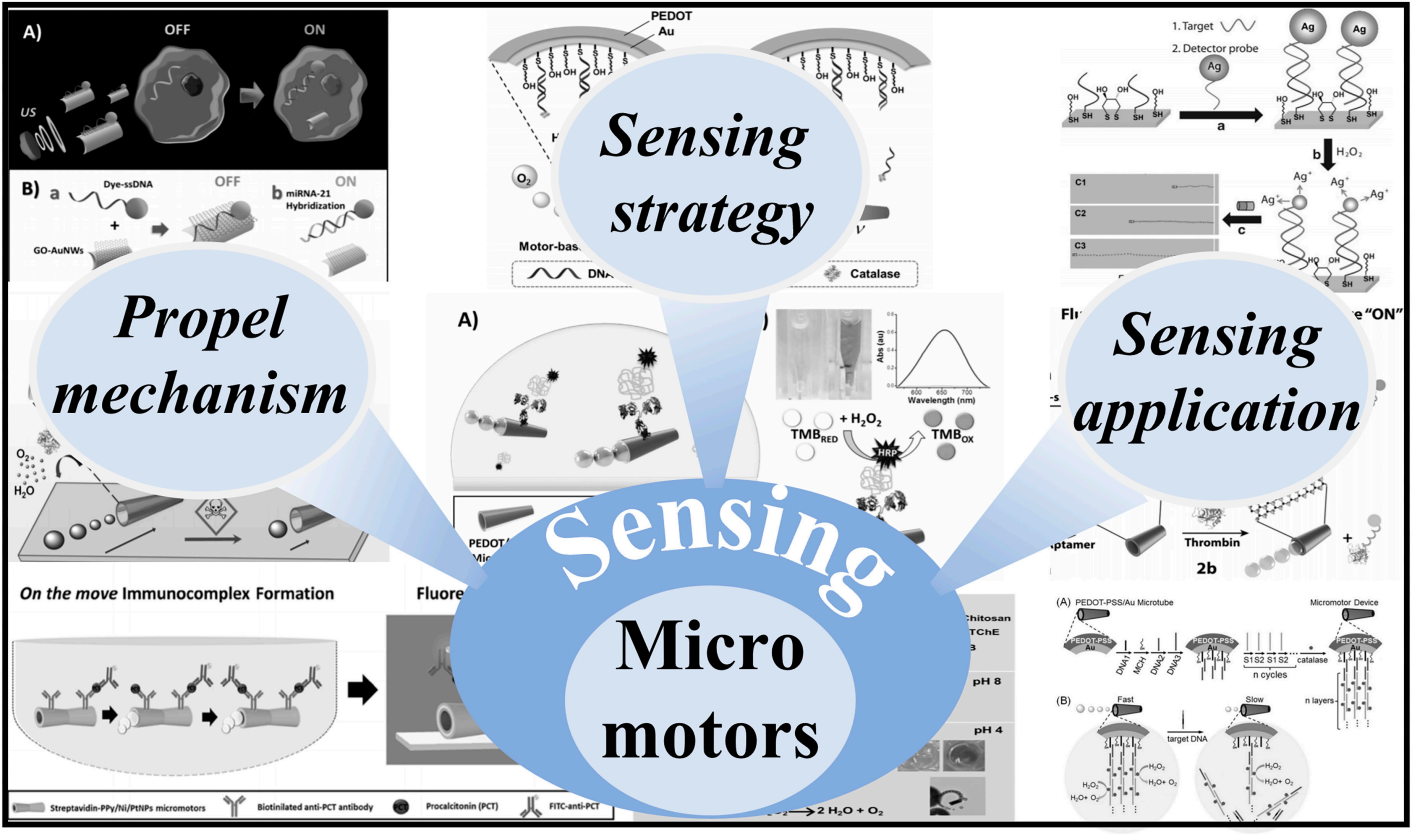
Scheme that describes the general flow and entire information of the review.

In this paper, we gave a review on the development of tailoring micromotors for sensing, involving their propulsion mechanism, sensing strategies, and applications (Fig. 1). First, the propulsion mechanism of micromotors was briefly summarized including free-based propulsion and fuel-free propulsion. Then, we focused on the sensing stratagems of micromotors including speed-based sensing strategy, fluorescence-based sensing strategy, and other strategies. After that, the applications of micromotor-based sensing platform in the field of environmental science, food industry, and biomedical area were introduced in detail. Finally, we discussed the challenges and prospects of the micromotors tailored for sensing. This comprehensive review was anticipated to facilitate readers’ understanding of micromotors for sensing and helped them to follow up the research frontiers in this field.

**Fig. 2.**
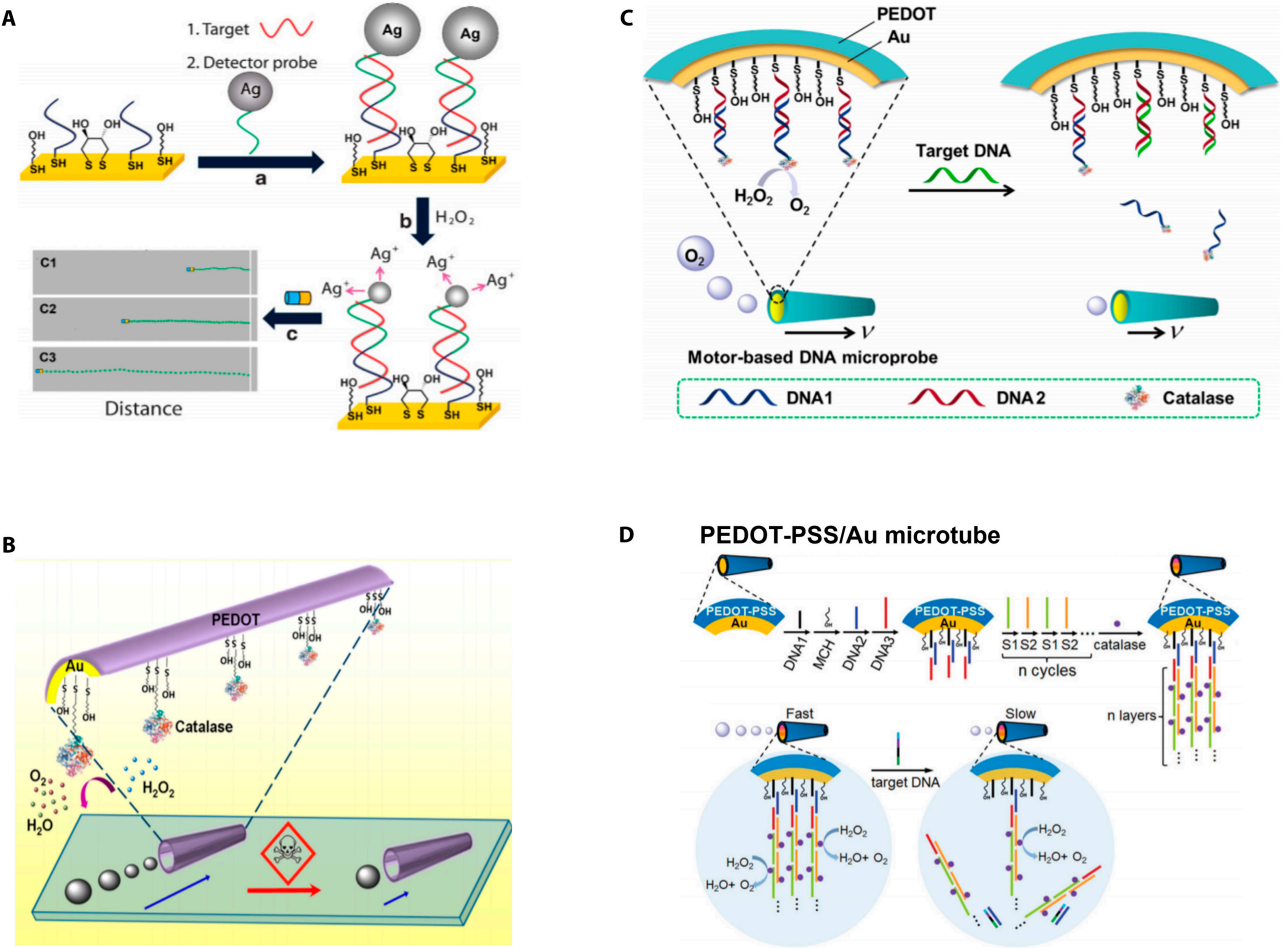
(A) Schematic illustration of velocity-based sensing involved with silver tags. Reprinted with permission from Springer Nature [65]. (B) Schematic illustration of velocity-based sensing based on the activity of catalase. Reprinted with permission from the American Chemistry Society [69]. (C) Schematic illustration of velocity-based sensing based on the amount of catalase. Reprinted with permission from Elsevier [70]. (D) Schematic illustration of velocity-based sensing involved with multiple catalase layers. Reprinted with permission from the Royal Society of Chemistry [71].

## Propulsion Mechanism of Micromotors

Due to their tiny size, micromotors usually work in the environment of low Reynolds number. Thus, external energy is needed for locomotion because the inertia cannot maintain the motion of micromotors due to the viscous resistance and Brownian motion. Propulsion mechanism, which the motion of micromotors relies on, is one of the principal issues in the research field of micromotors. Up to date, there have been copious mechanisms proposed for propelling micromotors [26–29]. Typically, the micromotors can realize movement based on chemical gradients or locally generated fields resulting from their reaction with the surrounding medium. However, these chemical-propelled micromotors usually resulted in incomplete fuel degradation, hindering the development of micromotors in biomedical engineering. To address the challenge, fuel-free micromotors, which are powered by external stimulus such as ultrasonic, magnetic, and light, come to the forefront as biocompatible mechanisms. This chapter will carry out a brief review on the propulsion mechanisms of micromotors involving fuel-based mechanism and fuel-free mechanism. In this section, readers fascinated by this aspect can be guided to specified reviews.

### Fuel-based mechanism

#### Self-electrophoresis

So far, a great number of micromotors have been produced based on self-electrophoresis. Notably, most of these micromotors are bimetallic. In this case, micromotors can realize self-propulsion via the self-generated electroosmotic flow in liquid environment resulting from chemical gradients [30]. In detail, the chemical reaction between micromotors and surrounding medium can lead to an asymmetric ion distribution around the micromotors. Then, the resultant electrolyte gradient can form a local electric field, thus propelling the micromotors. It is worth mentioning that their motion direction is dependent on the type of surface charge of the micromotor and the distribution of zeta potential. As a typical example, when micromotors composed of platinum/gold (Pt/Au) were exposed in the hydrogen peroxide (H_2_O_2_) environment, the consumption and oxidation of H_2_O_2_ occurred in the anode end (Pt) and cathode end (Au), respectively, resulting in an asymmetric proton distribution between Au end and Pt end [31]. This uneven proton distribution led to an electroosmotic flow from Pt to Au end, thus driving the micromotors to move. Apart from H_2_O_2_, chemicals such as hydrazine that can preferentially reduce or oxidize on one code of such bimetallic micromotors can be employed as fuels in the self-electrophoresis system [32,33]. Moreover, bio-electrochemical reactions can also be utilized in the self-electrophoresis system. Mano and colleagues [34] developed a kind of self-propelling carbon fiber based on the reaction of oxygen (O_2_) and glucose. By coating one end of the conductive carbon fibers with electrocatalyst for reducing O_2_ into water and the other end with bioelectrocatalyst for oxidizing glucose, uneven proton distribution was formed at the water–air interface. This anisotropic structure led to fast locomotion of the fibers, indicating that bioelectrochemical energy could be converted into propulsive force for driving micromotors. Despite that great progress has been made in the field of self-electrophoresis-based micromotors, there still remain several nonnegligible problems. In terms of their applications in analytical sensing, the major problem is that most fuels employed in this system are toxic, which might be harmful to the samples. In addition, the principle of self-electrophoresis indicates that it cannot work in the environment with high ion concentration. Besides, most micromotors based on self-electrophoresis exhibit low efficiency, which cannot meet the requirement of practical applications.

#### Self-diffusiophoresis

Micromotors based on self-diffusiophoresis can achieve self-driven locomotion through the asymmetric concentration gradient field. There are 2 classifications of self-diffusiophoresis mechanism: electrolyte and nonelectrolyte diffusiophoresis. In electrolyte diffusiophoresis, the molecules responsive for the formation of the concentration gradient are charged molecules. In this case, the charged molecules derived from the anodic and cathodic reactions diffuse at different speeds in the solution due to different diffusion coefficients, leading to a concentration gradient, which forms a diffusion-induced electric field for driving micromotors [35–37]. Notably, the electric field in electrolyte self-diffusiophoresis differs from that in self-electrophoresis. For self-electrophoresis, chemical reactions occur in the anode and cathode of micromotors. The resultant ionic products are enriched in different areas of the motor surface, forming a gradient electric field inside the motor, which is essentially an internal gradient field. For electrolyte diffusiophoresis, the anode and cathode reactions take place in the local space and the generated ions are locally distributed on one side of the motor. Due to the differentiation of different ion diffusion coefficients, ions diffuse outward at different migration speeds, forming a concentration gradient. This concentration gradient further generates a diffusion-induced electric field, which is essentially an external gradient field. Besides, the external charged substrate can have influence on the motion of micromotors based on electrolyte diffusiophoresis because there is also an electric field between the charge in the solution and the charge on the substrate surface. This electric field can form an electroosmotic flow. The motion of the micromotor is decided by the relative magnitudes of charges on external substrate and micromotors. Notably, micromotors based on gradients of hydrion (H^+^) or hydroxide (OH^−^) ions show best efficiency benefiting from the fast diffusion of H^+^ and OH^−^ [38]

On the contrary, in the nonelectrolyte diffusiophoresis, asymmetric catalytic reactions tend to occur for building nonelectrolyte concentration gradients. The asymmetric pressure gradient formed by the interaction between uncharged molecules and the micromotor acts as an osmotic force to drive the locomotion of micromotors. It is noteworthy that although micromotors based on nonelectrolyte diffusiophoresis normally exhibit lower efficiency than those based on electrolyte diffusiophoresis, they are able to work efficiently in high ionic aqueous environments. Besides, the motion direction of the micromotors driven by the nonelectrolyte concentration gradient is dependent on whether the interaction between the motor and the solute is repulsive or attractive. Overall, self-diffusiophoresis is a kind of propulsion mechanism of micromotors that derives from the concentration gradient of solutes, exhibiting high energy conversion efficiency (ECE).

#### Bubble propulsion

Due to the limited applicability of micromotors based on self-electrophoresis and self-diffusiophoresis in salt-rich solutions, bubble-driven micromotors are then proposed and have come to the forefront in the field of micromotors since the concept was conceived. Generally, the autonomous locomotion of bubble-driven micromotors is achieved by the recoil force derived from the generation of bubbles. Notably, the bubble propulsion is realized through both the microjets generated when the bubbles grow and collapse on the surface and the impulse generated by the bubbles detaching from the surface of the micromotor [39,40]. H_2_O_2_ solution is the most commonly used medium fuel in the system of bubble-driven micromotors. In this case, the micromotors are usually made from or composed of Pt or Au, which can act as catalase. With the catalysis, H_2_O_2_ can be decomposed into O_2_ and H_2_O. The excessive generation of O_2_ bubbles can create the recoil force for propelling the micromotors. It is worth mentioning that the velocity of micromotors is influenced by the radius of the bubbles and the frequency of the bubble generation, both of which are dependent on the concentration of the fuels. Although micromotors based on bubble propulsion have been widely used in various fields benefiting from their high efficiency, their further applications in biomedical areas are hindered by the potential toxicity of H_2_O_2._

Later, micromotors based on zinc (Zn) or magnesium (Mg) are proposed. This kind of micromotors can react with H^+^ in the aqueous solution, generating hydrogen (H_2_) bubbles to drive the micromotors [41]. Higher concentration of H^+^ is conducive to faster locomotion. Notably, this sort of micromotors can be applied to the in vivo environment. For example, Cai et al. [42] produced Mg-based micromotors that could move efficiently in the stomach because of the existence of gastric juice. Besides, a kind of biocompatible enzymatic micromotors based on urease was proposed by Patino et al. [43], which could decompose the urea into bubbles of ammonia and carbon dioxide (CO_2_). Although micromotors based on bubble propulsion can move efficiently, their motions are highly dependent on the surrounding mediums.

### Fuel-free mechanism

The micromotors based on decomposition of chemical fuels may lead to incomplete consumption of fuels, hindering their applications in biomedical fields. Thus, the trends in micromotor development then convert to the explorations of new external energy for propelling. As a result, researchers proposed fuel free micromotors that are driven by the external stimuli involving magnetic, light, or ultrasonic electric fields. Fuel-free micromotors show the advantages of remote control, long lifetime, and high biocompatibility, exhibiting broad application prospects in various fields. Magnetic fields, which have been widely used in remote control in diverse fields, also act as a promising power source for propelling micromotors. Basically, magnetic field can be employed to control the direction by integrating magnetic compositions into micromotors. The remote guidance of micromotors endows them great potential in different areas. Besides, magnetic fields are applied to drive micromotors without any external fuels. Dreyfus et al. [44] proposed a magnetic micromotor based on DNA chain. To be specific, the DNA chain was linked with colloidal paramagnetic beads, serving as a flexible artificial flagellum, which allowed propulsion of the micromotors under the external magnetic field. Notably, the direction and speed of these micromotors could be tuned by adjusting the frequency of the magnetic field. In addition, Zhang et al. produced a rotating nickel nanomotors purely propelled by magnetic field. With the control of uniform rotating magnetic field, the nanomotors exhibited a tumbling motion due to the spatial asymmetric doublets [45].

Apart from magnetic field, ultrasonic wave is also one promising tool for remote propulsion of micromotors. The first micromotor based on ultrasonic propulsion, which was composed of Au and ruthenium (Ru), was proposed by Wang et al. [46]. This kind of micromotor was propelled by the differential pressure field derived from the interference between 2 ultrasonic waves. In addition, the micromotors can also realize fuel-free locomotion triggered by electrical fields [47–49]. The most common mechanism of micromotors in this case is based on their electroosmotic properties in low-frequency electric fields. Also, there are rotary micromotors driven by the interaction between electric field of high frequency and electrically polarized nano-entities. Overall, magnetic, ultrasonic, or electric field-driven micromotors exhibit the advantages of high energy penetration, strong propulsion, and relatively small requirements for the surrounding medium, but the manufacturing process and equipment parameters are complex. Furthermore, these propulsion mechanisms rely on device of high-energy external fields.

On the contrary, light, which is controllable and renewable, also serves as one of the most common external fields in remote propulsion of micromotors [50–52]. As the parameters of light, such as light intensity, light frequency, and polarization degree, can be easily and precisely tuned, the light-driven micromotors hold the advantages of high controllability, good programmability, and easy operation. It’s worth mentioning that the start and stop of the motion of the light-driven micromotors can be quickly adjusted by the on/off mode of the light source. Furthermore, their motion performance is affected by the incident light intensity along with the type and concentration of the solution substance. Generally, the motion of micromotors triggered by the light stimulus originates from the asymmetric structure, the asymmetric concentration gradient field, or the non-equilibrium hydrodynamic force established under the non-uniform illumination condition. Breaking the symmetry of the pressure distribution is the principle of driving the motors. By way of example, micromotors driven by light can achieve autonomous locomotion through photothermal mechanism [53,54]. In other words, light-induced asymmetric temperature gradients can drive micromotors. By utilizing photothermal materials to absorb and convert light energy into heat energy, a temperature gradient can be built around the micromotors. Because the thermophoretic forces cancel each other out due to the symmetrical thermal diffusion, the photothermal particles with symmetrical geometry usually behave as Brownian under illumination. Therefore, most of the micromotors driven by light-induced temperature gradients possess asymmetric geometries with one side of photothermal effect material and the other side of non-photothermal effect material. Under continuous light stimulation, a temperature gradient is generated gradually. The water flowing in the low temperature part of the micromotor can flow around its surface to the high temperature part, and the reaction force of the water flow drives the micromotor.

Notably, these fuel-free propulsion mechanisms can be integrated to the fuel-powered micromotors to impart them with multifunction. Typically, the magnetic propulsion mechanism can be introduced to the fuel-powered micromotors to realize remote control of their motion direction for meeting different requirements [55–57]. Very recently, Zhao et al. [58] incorporated the light-driven mechanism with bubble propulsion for achieving the remote speed manipulation. More explicitly, these motors were Janus structures with one side of glucose catalase and the other side of Au, which could catalyze glucose and generate thermal gradient with the near-infrared (NIR) irradiations, respectively, resulting in opposite driving force. Thus, the motion performance can be easily tuned by adjusting the NIR light irradiation. In conclusion, fuel-free micromotors propelled by the external stimuli such as magnetic, light, or ultrasonic electric fields exhibit predominance of remote control, long lifetime, and high biocompatibility, broadening the applications of micromotors.

## Stratagems of the Sensing Micromotors

Benefiting from various propulsion mechanisms, micromotors can realize efficient autonomous locomotion in the surrounding medium. Notably, the property of self-driven movement makes micromotors promising in serving as sensors for bioassays because the dynamic movement can realize efficient mixing of the sample solution, thus improving the probe–target interactions, which is conducive to the increase of the detection sensitivity and reduction of analysis time. In addition, benefiting from their tiny size, micromotors can even work in the sample solution of low volume, which is highly relevant in daily clinical samples. Furthermore, micromotors enable the in situ detection, which can work in the raw samples, simplifying the beforehand operation and reducing the assay cost. Attracted by these aspects, scientists have paid much attention in tailoring micromotors for bioassays.

On the one hand, micromotors have been introduced to assays for assisting the assays toward higher efficiency due to their inherent autonomous movement. For instance, Morales-Narváez et al. [59] applied the micromotors to the biosensing system to assist the transportation of the targets. As these bubble-propelled micromotors could move autonomously, they were capable of translating the surrounding fluid convection into general vortex effect for non-invasive and continuous mixing. Such function greatly expedited the interaction between the probes and targets, thus enhancing the performance of the detection platform. In addition, Restrepo-Pérez et al. [60] executed catalytic micromotors in the process of sample pre-concentration that had shown great importance in achieving highly sensitive detection. To be specific, boundary with chevron and ratchet shapes was designed to trap micromotors that were pre-functionalized with streptavidin for selective capture of the targets. By this, targets could be efficiently transported and concentrated without any external energy source, thus facilitating the miniaturization and integration. Recently, the target molecular enrichment assisted by micromotors was also utilized to realize ultrasensitive surface-enhanced Raman scattering sensing [61]. Fan et al. successively proposed micromotors based on bubble propulsion and magnetic propulsion to enrich targets from the samples because the autonomous locomotion enabled effective contact between analytes and micromotors, consequently resulting in promoted Raman intensity. More recently, Hou and coworkers employed bubble-propelled MnFe_2_O_4_-based micromotors with property of adsorption to improve the solid-phase extraction [62]. Benefiting from the continuous locomotion of micromotors, the adsorption was enhanced, which was conducive to the increase of the detection sensitivity. More intriguingly, they proved that the autonomous movement also prevented the micromotors from depositing, thus exhibiting efficient bubble generation and excellent detection ability. Overall, the autonomous movement endows micromotors with the ability to promote the sample mixing and enhance mass transfer, providing valuable reference for meliorating detection sensitivity, simplifying the operation, and reducing the assay time.

On the other hand, micromotors themselves can serve as sensors in the assays. In this case, micromotors are able to transmit the signals of the recognition events into visualizable analytical signals based on different sensing stratagems as well as enhancing the mass transfer. Intriguingly, extensive signals can be detected simultaneously in one sensing assay because each micromotor can act as a single sensor, which promotes the sensing reproducibility and accuracy, reduces the assay cost, and simplifies the operation. In the following section, we will introduce the stratagems of the sensing micromotors including speed-based sensing strategy and fluorescence-based sensing strategy, cover their working principles, and list the corresponding typical examples.

### Speed-based sensing strategy

The velocity of micromotors, one visualizable characteristic parameter of the locomotion behavior, can be directly observed with the assistance of optical microscopy. Due to the responsiveness of the velocity to the environment, the velocity acts as a unique visible signal in the micromotor-based sensing strategy, opening a novel paradigm in sensing fields. For example, Lei et al. found that the viscosity of surrounding solution had influence on the motion performance of micromotors, which inspired them to present a novel micromotor for viscosity detection. To be specific, the locomotion velocity decreased with the increasing of ambient viscosity. Thus, by recording the relationship between velocity and viscosity, the sensing of viscosity could be realized [63]. In addition, as mentioned above, for the fuel-powered micromotors, their speeds can be influenced by the concentration of the fuels in surrounding solution. In other words, the speed of micromotors can reflect changes of the fuel concentration in the solution, thus serving as a critical visualizable signal in detecting the concentration of fuels. Usually, the relationship of the velocity and the fuel concentration is recorded in advance, based on which the concentration of sample solution can be calculated by observing the motion velocity of micromotors. The first speed-based sensing micromotor system was proposed by Wang and colleagues [64]. They found that the presence of silver ions exhibited selective and sensitive influences on the velocity of micromotors based on self-electrophoresis. To be specific, the speed of these motors was positively related to the concentration of silver ions. By using the optical microscope to track the variation of the velocity, the concentration of silver ions could be detected. Based on this work, they further proposed a novel kind of micromotors for detecting targets in a sensitive and quick way, indicating the great potential of the motion-based signal transduction in sensing application, as shown in Fig. 2A [65]. To elaborate, the motors were modified with specific probes for capturing targets. Then, specific silver tags were added to the system to form sandwich hybridizations. As the silver ions had influence on the velocity, the number of targets captured by the motors could be quantified by measuring the velocity changes of motors or the aggregate traveling distance of the motors. Later, Gao et al. [66] proposed hybrid micromotors with palladium (Pd) coating on one side of aluminum (Al) particles, which could move spontaneously in multiple solutions involving H_2_O_2_, acid, and base. As these micromotors were powered by the bubbles resulting from the reaction between themselves and surrounding solution, the locomotion performance of those micromotors was mainly dependent on the concentration of the surrounding solution. Thus, the velocity of micromotors served as a direct signal for sensing the concentration of the sample solution.

Apart from the concentration of fuels, the velocity is also related to the weight of micromotors. Specially, molecules captured by the micromotors will lead to the weight increase. Thus, velocity variation resulting from weight changes acts as a promising sensing strategy. However, considering that the weight variation resulting from the mass loading is weak, the sensing system in this strategy is usually insensitive. Thus, exquisite designed labels are introduced. By way of example, Yu et al. [67] designed a fast micromotor-based sensing platform for detecting protein targets with the help of glycidyl methacrylate microspheres, which were modified with the secondary antibodies. The targets captured by the probes modified on the motor surface could also have selective recognition with the secondary antibodies, which increased the weight of micromotors, thus slowing down the motion velocity. In other words, in this case, the velocity of micromotors was negatively correlated with the concentration of targets. It was proved that this method enabled fast and sensitive detection of proteins, demonstrating great potential in sensing applications. More recently, Shafiee and colleagues [68] proposed a sensing platform based on micromotors and loop-mediated isothermal DNA amplification. The presence of targets would trigger the amplification of nucleic acid sequences, and the large-sized amplicons then slowed down the locomotion of the micromotors.

For micromotors based on catalyst-assisted propulsion, their motion performance is also relative to the activity and amount of the catalyst. Higher activity of catalyst results in better motion performance; thus, higher velocity is observed. On the basis of this principle, Wang and colleagues developed a biocatalytic bubble-propelled micromotor for sensing [69]. As shown in Fig. 2B, the catalyst was initially decorated on the micromotors for propelling. Because the attendance of targets could induce the inhibition of catalyst, the bubble generation that resulted from the catalytic reaction was reduced, which indirectly slowed down the motion velocity. Thus, quantification of targets could be realized by measuring the velocity of micromotors. In addition, the amount of catalyst is also unignorable to the motion performance of such micromotors. Attracted by this fact, scientists have come up with diverse strategies. As a successful example, Ju and colleagues [70] presented biocatalytic bubble-powered micromotors with catalase layer assembled in the inner surface of the tubular micromotors with the help of DNA conjugation (Fig. 2C). With the attendance of target sequences, these catalases would be released via DNA strand replacement hybridization, leading to the reduction of the catalases, which decreased the velocity. By observing the motion speed through optical microscope, they found that the velocity was negatively correlated with concentration of targets. It was worth mentioning that this approach showed better sensitivity because the engine section and sensing element were arranged on the same part, which meant that the capture of targets had direct influence on the driving force of the micromotors. However, as one target could get rid of only one catalase, the sensitivity of this strategy could be further improved. Aiming at this, Ju and colleagues [71] replaced the single catalase layer with multiple catalase layers, further developing an improved platform for sensitive DNA sensing. Here, multilayer DNA strands were assembled inside the tubular micromotors by cyclic alternate hybridization assembly to load multiple catalase layers, as shown in Fig. 2D. Notably, benefiting from the multiple catalase layers, this improved micromotor could move efficiently even in fuel solution. Once the targets attended, the DNA chains with multiple catalase layers were released. The removal of multiple catalase layers triggered by only one target resulted in an obvious decrease in the motion speed, implying excellent sensitivity of this speed-based sensing strategy. Later, to overcome the inefficient biorecognition inside the tubular micromotors, they applied such a sensing strategy to the jellyfish-like micromotor [72]. Benefiting from the large open sensing surface, this newly proposed platform showed higher sensitivity for detecting DNA.

Generally speaking, apart from enhancing the mass transfer, the micromotors can also act as mobile sensors for converting recognition events to readable signals. The velocity, which is an important parameter reflecting the locomotion performance of micromotors, is considered as a useful readable signal. A diversity of speed-based micromotor sensing systems has been developed. It has been proved that those speed-based systems enable sensitive detection to obviate the need for cumbrous optical components and exquisite apparatus, which ushers in a new era for the analytical sensing field. However, there are still some restrictions on the type of targets in the motion-based micromotor sensing systems. For these targets that can be detected on the basis of the influence of mass loading on the velocity, their weights must achieve a certain level for efficient influence on the velocity. In addition, only targets that are capable of affecting the activity or the amount of catalase can be detected via the catalase-related speed-based micromotor sensing systems. Thus, scientific endeavors are also devoted to the development of other sensing strategies based on micromotors.

### Fluorescence-based sensing strategy

Fluorescence-based strategy, which is the most common strategy in traditional sensing fields, has also been incorporated with the micromotor platform for innovative sensing methods. Fluorescence is the light emitted by a substance when it absorbs light or other electromagnetic radiation, which possesses dominance of easy readout by naked eyes. By integrating the fluorescence-based sensing strategy with the micromotors, which enables highly sensitive detection of raw sample of low volume in short time as mentioned above, a powerful platform for sensing is provided with easy operation, remarkable sensitivity, outstanding selectivity, and high accuracy. According to the transition pattern of the fluorescence signal, the fluorescence-based micromotor sensing strategy can be categorized into “off–on,” “on–off,” and other detection strategies.

**Fig. 3.**
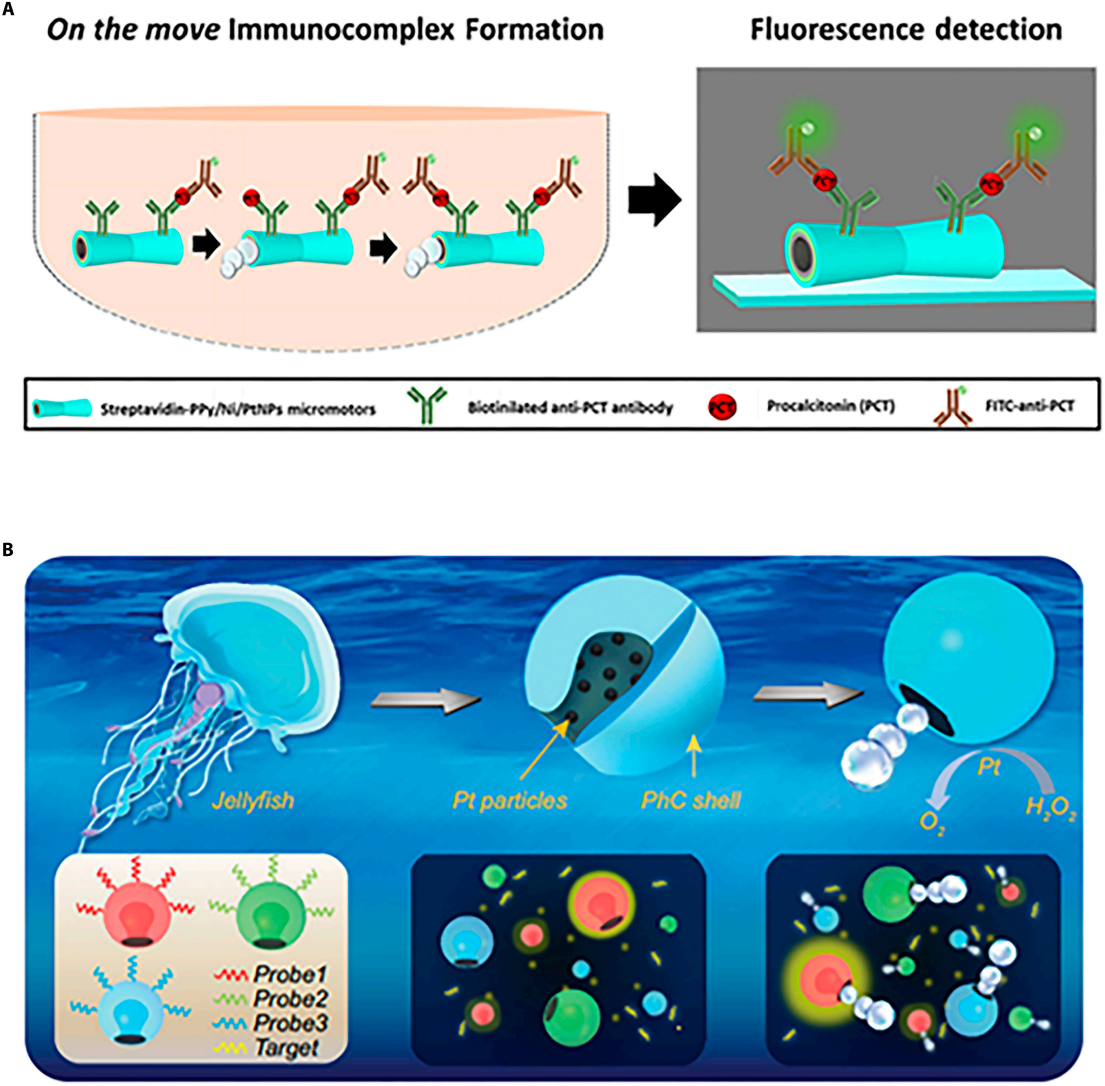
(A) Schematic illustration of micromotor-based fluorescence immunoassay. Reprinted with permission from the American Chemistry Society [73]. (B) Schematic illustration of micromotors with characteristic structural colors for the multiplex assays. Reprinted with permission from Oxford Academic [75].

As we can infer from the name, the fluorescence signal of “off–on” strategy goes through a process from “off” with the attendance of the targets to “on” with the presence of the targets. A typical “off–on” detection strategy is based on immunofluorescence technique. In this case, the micromotors are usually decorated with antibodies for capturing targets. With the help of fluorescent labels, the event that the targets are captured can be read out through the fluorescent signals. For example, Escarpa and coworkers [73] developed a novel micromotor-based fluorescence immunoassay, where the specific antibodies were modified on the surface of micromotors, as depicted in Fig. 3A. Notably, the initial system showed no fluorescence. When exposed to the sample solution, the micromotors moved spontaneously, enhancing the likelihood of antibody–target contacts. After being captured by the antibodies on the micromotor surface through specific recognition, the targets were then labeled with the fluorescent labels, exhibiting readable fluorescent signal. It was worth mentioning that the intensity of fluorescent signal grew with the increase of the label captured, which indirectly indicated the amount of targets. Thus, the quantification of targets could be realized by observing the intensity of fluorescent signal. Compared with traditional static immunofluorescence assays, the platform enabled detection in clinical sample of smaller volume with desirable sensitivity benefiting from the autonomous movement of micromotors. Considering the great potential of the surface modification of micromotors, fluorescence-based nucleic acid-specific recognition can also be integrated with micromotors by immobilizing probes on the surface for sensing. With the specific recognition among probes, targets, and fluorescent labels, the amount of target sequence can be obtained by observing the intensity of the fluorescent signal. Based on this principle, Oksuz and colleagues [74] decorated the W_5_O_14_-composed micromotors with “off–on” sensing probes. Intriguingly, in this work, apart from the fluorescence signal, changes in velocity that responded to the targets could also be detected, providing a dual-responsive sensing detection.

It is worth mentioning that multiplex detection could be realized by integrating the non-fluorescent barcodes into the micromotor sensing system. As an interesting example, Cai et al. [75] proposed an ingenious kind of bubble-propelled micromotors with characteristic structural colors for the multiplex assays (Fig. 3B). To be specific, these micromotors were derived from the stomatocyte-like silica photonic crystal templates that were imparted with obvious characteristic structural colors because of the photonic band gap. By employing silica nanoparticles with different sizes as templates, micromotors with diverse structural colors could be fabricated. Pt nanoparticles were loaded into the cavity of the stomatocyte-like microcarriers to obtain bubble-propelled micromotors. For multiple detection, micromotors with different structure colors were decorated with different probes. With the selective recognition between nucleic acid, fluorescent signals occurred on the micromotors whose immobilized probes coupled with corresponding targets. Thus, the multiplex qualitative detection of different targets could be realized by combining the fluorescence signals with the structural colors. This platform allowed for simple but efficient multiplex detection, indicating great potential in sensing fields. The specific probes functionalized on the surface of micromotors for capture of specific target might limit the detection ability of micromotors for detecting other targets. To overcome this defect, Park and Yossifon [76] designed a generic metallodielectric Janus micromotor to carry out the detection of diverse targets through combination of different functionalized beads, enhancing the repeatability of micromotors for detecting different kinds of targets. These Janus micromotors could be incorporated with functionalized beads through dielectrophoresis. To elaborate, the beads could be attracted to or repulsed from the micromotors by adjusting the electric field frequency according to their property or geometry. The probes immobilized on the beads could realize capture of targets, which could be read out via the fluorescence signal. Thus, these micromotors could be utilized to detect diverse targets by combining with different functionalized beads. This platform overcame the limitation of single target detection of traditional micromotors, opening up novel realizations of general sensing strategy.

**Fig. 4.**
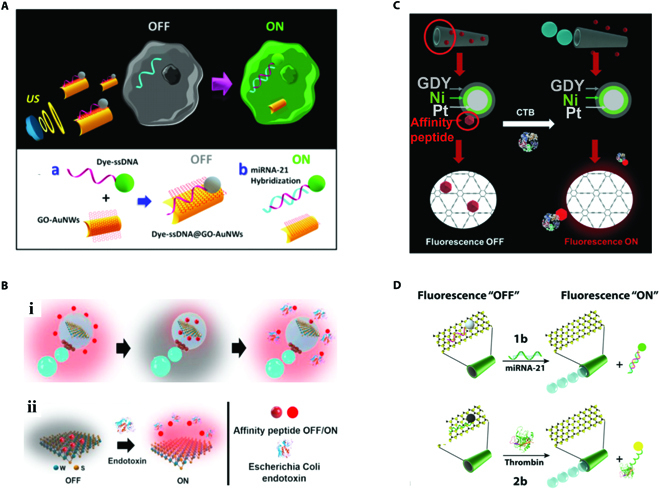
(A) Schematic illustration of “off–on” fluorescent sensing strategy based on the quenching property of GO on fluorescent probes. Reprinted with permission from the American Chemistry Society [82]. (B) Schematic illustration of WS_2_-composed micromotors for “off–on” fluorescent sensing strategy. Reprinted with permission from Elsevier [85]. (C) Schematic illustration of “off–on” fluorescent sensing strategy based on the quenching property of graphdiyne on fluorescent probes. Reprinted with permission from the Royal Society of Chemistry [79]. (D) Schematic illustration of MoS_2_-composed micromotors for “off–on” fluorescent sensing strategy. Reprinted with permission from John Wiley and Sons [83].

Another “off–on” detection strategy of micromotors relies on the property of fluorescence quenching of specific elements such as carbon-based nanomaterials and transition metal dichalcogenides. Graphene oxide (GO) is a new type of carbon-based nanomaterial with excellent properties of specific surface area and abundant functional groups on the surface [77,78]. The distinct surface properties of GO enable integration of diverse receptors, providing a marked clue for designing micromotors for fluorescent-based sensing. To be specific, these GO nanomaterials can lead to the quenching of fluorescence because they are able to adsorb the fluorescent labels via interactions involving hydrophobic interactions, π-stacking interactions, electrostatic effects, ion exchange, hydrogen bonding, and so on, resulting in the fluorescence resonance energy transfer (FRET) effect. Due to the FRET effect, the fluorescence is quenched, exhibiting an “off” state. However, the fluorescence can be recovered when the labels are removed away from the surface of GO nanomaterials. By combining this mechanism with the micromotors, “off–on” detection strategies are proposed [79–81]. By way of example, Wang and colleagues [82] proposed GO-composed micromotors, which could adsorb dye-labeled probes through π-stacking interactions. Due to the fluorescent quenching mechanism of GO on the dye-labeled probes, the initial micromotors with dye-labeled probes exhibited little fluorescence, as shown in Fig. 4A. Once they were exposed to the solution containing targets, the dye-labeled probes preferentially bounded with the target sequences, leaving away from the GO surface, which led to the immediate recovery of fluorescence. This approach enabled the direct screening of targets through the “off–on” fluorescent signal.

Besides, 2-dimensional (2D) molybdenum disulfide (MoS_2_), a kind of transition metal dichalcogenides with large surface area-to-volume ratios, has been utilized to realize the “off–on” sensing. In 2016, Wang and colleagues [83] combined the MoS_2_ with micromotors for the first time, yielding a novel kind of MoS_2_-based tubular micromotor. Similar to the GO, the MoS_2_ on the surface of micromotors is capable of adsorption of fluorescent probes and cause fluorescence quenching due to the FRET effect (Fig. 4D). After the probes were recognized by targets, they were released and then their fluorescence was recovered. By measuring the intensity of fluorescence, the concentration of targets could be calculated. Notably, compared with GO, MoS_2_ possessed dominance of direct aqueous dispersion and facile high-throughput synthesis, benefiting from its distinct trilayer-type architecture, which was more ideal for practical application in sensing field. In addition, recent studies have indicated that tungsten disulfide (WS_2_) showed strong adsorption of peptide because its negative charge derived from the surface sulfide groups could interact with the peptide with positive charge through hydrophobic and electrostatic interactions [84,85]. Based on the same mechanism, Escarpa and coworkers [85] proposed a WS_2_-based micromotor and compared its sensing performance with MoS_2_-based micromotor. As depicted in Fig. 4B, the fluorescence of probes quenched when attached to the WS_2_ and recovered when specific recognition occurred. It was found that WS_2_-based micromotor sensing system showed higher sensitivity because the rougher surface area provided larger available surface area for probe adsorption. Furthermore, it was proved that such “off–on” micromotor sensing system could maintain its sensing stability and remarkable analytical performance for over 2 months, indicating great potential in practical sensing assays. Besides, its also reported graphdiyne, a kind of 2D nanomaterials, could be integrated into the micromotor system for “off–on” sensing due to their absorption ability of fluorescent labels, as shown in Fig. 4C [79,86]. Moreover, graphdiyne conferred the micromotors with rough surface, enlarging the reaction area for label adsorption, which indirectly increased the sensitivity of the detection and reduced the detection limit. In general, the “off–on” micromotor-based sensing strategy derived from fluorescence quenching mechanism greatly advanced the development of micromotors in sensing fields.

Apart from “off–on” sensing strategy, “on–off” sensing strategy derived from the fluorescence quenching mechanism also served as an efficient sensing strategy in the area of micromotors. In this case, the micromotors are usually modified with fluorescent materials, which means that initially the micromotors themselves exhibited fluorescence signals arising from the fluorescent materials. The fluorescence quenches with the attendance of targets, which can lead to the fluorescence quenching of the fluorescent materials. The fluorescence goes through the process of “on” to “off,” serving as a detectable signal for sensing. Intriguingly, the efficient micromotors coupled with elaborate fluorescence “on–off” strategy can be utilized to confirm the attendance of targets rapidly. In 2015, Wang and coworkers [87] proposed the first micromotor-based fluorescent “on–off” detection approach by using the quenching phenomena of fluorophore fluoresceinamine with the presence of phosphoryl halides. In another work, Liu and colleagues [88] conceived europium metal organic framework (Eu-MOF)-based fluorescent micromotor for sensing. Eu-MOFs are porous materials with periodic network structure formed by self-assembly of rare earth elements as metal nodes and organic ligands. They serve as highly potential efficient fluorescent probes with strong anti-interference ability for sensitive detection. Based on the fluorescence quenching mechanism of ferric iron (Fe^3+^) on Eu-MOF, which was derived from the electron transfer, competitive absorption, and collapse of the framework, the Eu-MOF-based fluorescent micromotors allowed for “on–off” detection of Fe^3+^.

**Fig. 5.**
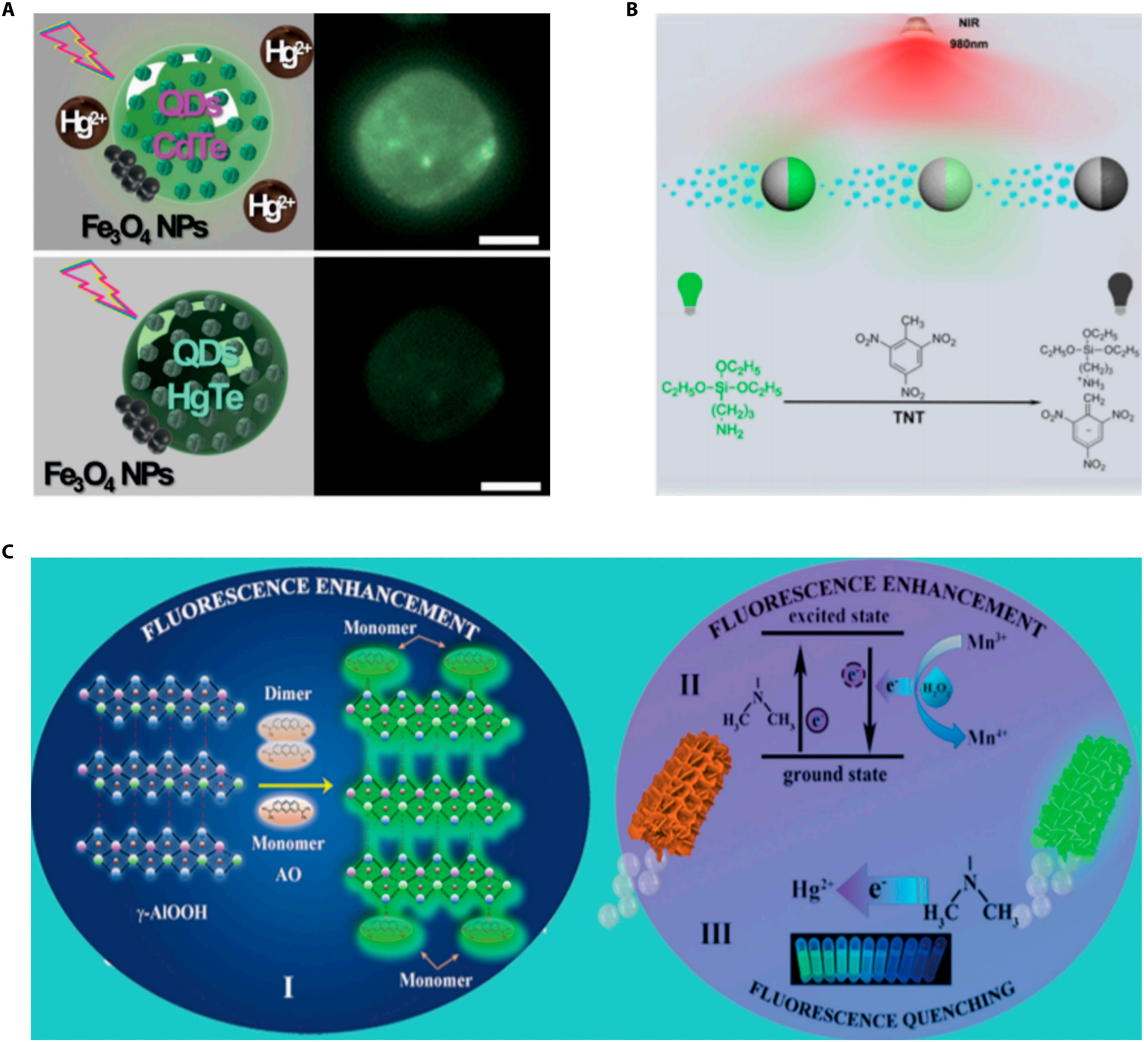
(A) Schematic illustration of CdTe-composed micromotors for “on–off” fluorescent sensing strategy. Scale bar, 20 nm. Reprinted with permission from John Wiley and Sons [50]. (B) Schematic illustration of “on–off” micromotor sensing system based on the chemical recognition between the TNT and UCNPs. Reprinted with permission from the Beilstein-Institut [92]. (C) Mechanism and schematic illustration of AO-composed micromotors for “on–off” fluorescent sensing. Reprinted with permission from the Royal Society of Chemistry [93].

It is worth mentioning that the fluorescence quenching mechanism of quantum dots (QDs) provides significant clues for designing micromotors for “on–off” sensing. Graphene quantum dots (GQDs) are an important graphene derivative with inherent fluorescence. Escarpa and colleagues [89,90] took advantage of the phenomenon that the endotoxin could lead to the fluorescence quenching of GQDs to develop a highly efficient micromotor-based detection. By immobilizing the GQDs with specific recognition receptors, the GQDs could bind with endotoxin, resulting in the formation of covalent cross-linking GQDs and contributing to the efficient fluorescence quenching strategy. The autonomous movement of micromotors was conducive to a quicker quenching, and the quenching extent was directly proportional to the concentration of targets, which indicated the great potential of the GQDs in serving as a powerful sensing platform. In addition, it has been proven that some metal ions can quench the fluorescence of QDs as well. For instance, the fluorescence of ZnS·Mn QDs can be quenched by copper (Cu^2+^) because the energy transformation from ZnS to Mn is blocked by Cu^2+^ through non-radiative recombination. Harnessing the advantages of this, Wang et al. [91] designed an “on–off” micromotor-based method for convenient monitoring of the Cu^2+^ concentration. Recently, the cation exchange ability of Zn-based and Cd-based QDs has also attracted much scientific attention in the field of micromotor-based sensing because the generation of novel nanostructures resulting from the cation exchange could lead to a fluorescent “on–off” process [50,79]. By integrating this mechanism with micromotors, Escarpa and colleagues [50] presented CdTe or CdSe@ZnS QD-based micromotors for selective “on–off” mercury detection. As demonstrated in Fig. 5A, the mercury ions (Hg^2+^) took the place of chromium ion (Cd^2+^) to generate HgTe QDs that exhibited no fluorescence, resulting in the fluorescence quenching of the initial QDs.

Besides, it is reported that some toxins such as 2,4,6-trinitrotoluene (TNT) could result in the fluorescence quenching of upconverting nanoparticles (UCNPs), based on which He and colleagues [92] proposed micromotors functionalized with UCNPs for sensitive “on–off” detection. In this system, the chemical recognition between the TNT and UCNPs brought about the formation of Meisenheimer complex that could strongly adsorb the emission spectrum of UCNPs, as shown in Fig. 5B. The “on–off” phenomenon took place because of the FRET effect from the UCNPs to Meisenheimer complex, serving as an important clue for visible detection of TNT. It is also reported that Hg^2+^ could contribute to the fluorescence quenching of the micromotor decorated with acridine orange (AO) due to the charge transfer from –N(CH_3_) in AO to Hg^2+^, which provided a useful clue for designing micromotors for sensing [93]. By integrating this strategy with micromotor system, the quantification of Hg^2+^ could be achieved by observing the extent of fluorescence quenching of AO. The mechanism of this sensing platform was presented in Fig. 5C. Generally speaking, the “on–off” detection strategy of micromotors enables quick visible detention. Furthermore, the fluorescence quenching phenomenon provides intensive design space for tailoring micromotors for biosensing.

**Fig. 6.**
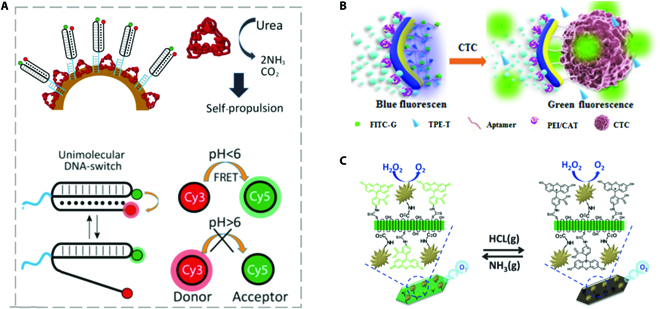
(A) Schematic illustration of micromotor immobilized with FRET-labeled DNA-based nanoswitches for sensing based on fluorescence color variation. Reprinted with permission from the American Chemistry Society [43]. (B) Schematic illustration of micromotor containing FITC and TPE for sensing based on fluorescence color variation. Reprinted with permission from Elsevier [94]. (C) Schematic illustration of fluorescence-based sensing strategy depending on the intensity of fluorescence. Reprinted with permission from the Royal Society of Chemistry [95].

In addition to the “off–on” and “on–off” fluorescence-based sensing strategy, the color change of fluorescence also acts as an efficient visible signal in micromotor-based sensing fields. By way of example, Patino et al. [43] proposed a sort of micromotor immobilized with FRET-labeled DNA-based nanoswitches, which could exhibit fluorescence in different colors in response to the different pH value. As shown in Fig. 6A, the DNA nanoswitch was single-stranded DNA in triplex structure with a DNA hairpin in the loop of which cyanine-3 fluorophore (Cy3) was linked. In addition, at the 3′-end of DNA portion, cyanine-5 fluorophore (Cy5), which served as an accepter to accept the resonance energy from Cy3, was conjugated. Notably, the formation of this unique triplex-forming structure was due to the collective effect of Watson–Crick interaction for DNA hairpin and parallel Hoogsteen interaction for the other sequence to the hairpin. Owing to the pH dependence of Hoogsteen interaction, the triplex form could be destabilized to a duplex structure when the pH value was over 6, resulting in the separation of Cy3 and Cy5, therefore hindering the FRET effect from Cy3 to Cy5. Based on this mechanism, the real-time pH value could be monitored by observing the variation of FRET signal of the micromotors. In another work, a Janus micromotor was presented for sensing based on the fluorescence color change strategy [94]. More explicitly, as shown in Fig. 6B, the micromotors were decorated with aptamers labeled with fluorescein isothiocyanate (FITC) and tetraphenylethene (TPE) derivatives. Intriguingly, with the competitive binding of targets, FITC and TPE were released from the aptamers, relieving the aggregation-caused quenching of FITC and the aggregation-induced emission of TPE, which decreased the emission of TPE and restored the fluorescence of FITC. Thus, the capture of targets could be sensed by employing the ratiometric fluorescence changes as a visible signal. Besides, the change of fluorescence intensity provides possible options for sensing (Fig. 6C) [95]. FITC, a common fluorescein, has 4 protolytic forms including dianionic, neutral, cationic, and anionic forms, based on which the fluorescence intensity varies according to the surrounding pH. By integrating the FITC molecules with micromotors, a pH-sensitive micromotor was designed. More explicitly, the fluorescence intensity enhances with the increase of pH. This innovative combination of materials in different fluorescent mechanisms provides various feasible strategies for micromotor-based sensing.

To summarize, fluorescence-based sensing strategies have attracted much scientific attention benefiting from their fast responsiveness, desirable sensitivity, and easy readout by naked eyes. A variety of traditional fluorescence-based sensing strategies including fluorescence-labeled nucleic acid recognition, fluorescent protein affinity, and fluorescence quenching mechanism have provided infinite design inspiration for tailoring functional micromotors for sensing, resulting in the generation of a large number of micromotor-based sensing approaches. Basically, the targets can be detected by observing the variation of the fluorescence signal because the attendance of targets usually leads to the fluorescent signal variation. Intriguingly, the combination of micromotors with different fluorescence-based strategies enables faster, more sensitive, and more accurate sensing as the motion performance of micromotors greatly enhances the mass transfer and sample mixing.

### Other sensing strategy

Apart from the typical velocity-based and fluorescence-based sensing strategies, there are also some other sensing strategies integrated with micromotors that are worth mentioning. In terms of bubble-propelled micromotors, the number of generated bubbles can also act as a visible signal of the surrounding reactions. Mattrey and colleagues [96] presented Pt-composed micromotors to quantify the surrounding H_2_O_2_ through the ultrasound imaging of expelled O_2_ bubbles. Ultrasound can detect single bubble because it elicits and identifies specific nonlinear oscillations of microbubbles with specialized pulses to eliminate most background signals. With the help of ultrasound detection, the detection limit of generated bubbles was 25 times lower than locomotion observation via microscopy, which even allowed the detection of trace H_2_O_2_ in vivo.

**Fig. 7.**
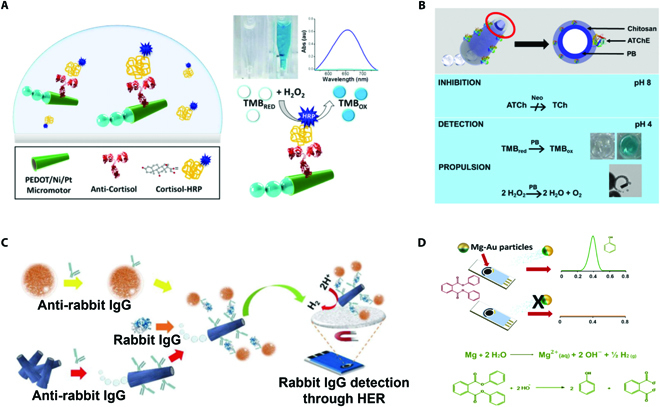
(A) Schematic illustration of colorimetric micromotor sensing system based on the TMB/H_2_O_2_ and HRP tags. Reprinted with permission from Elsevier [97]. (B) Schematic illustration of colorimetric micromotor sensing system based on TMB/H_2_O_2_ and Prussian Blue. Reprinted with permission from the American Chemistry Society [98]. (C) IrO_2_/Pt-composed micromotors for sensing based on electrochemical reaction. Reprinted with permission from John Wiley and Sons [100]. (D) Mg/Au Janus micromotors for sensing based on the electrochemical reaction between micromotors and targets. Reprinted with permission from the American Chemistry Society [99].

Micromotor sensing strategy based on colorimetric assays serves as a promising platform because the signal of colorimetric assays is naked eye visible. Wang and coworkers [97] reported micromotors for rapid naked eye detection by employing the system of 3,3′,5,5′-tetramethylbenzidine (TMB)/H_2_O_2_ and horseradish peroxidase (HRP) tag, considered as a typical colorimetric system. As shown in Fig. 7A, the antibody on the surface of micromotors could recognize with the tags with HRP or other appropriate peroxidases. Catalyzed by the HRP or other appropriate peroxidases, TMB could be oxidized and then generated soluble blue product, which acted as the naked eye visible signal of colorimetric assay. As they tagged the targets with HRP, the measurement of targets was realized by observing the color of the sample solution. More recently, on the basis of the same colorimetric mechanism, Escarpa and colleagues [98] incorporated Prussian Blue, an appropriate peroxidase, into the micromotor sensing system based on TMB/H_2_O_2_, exhibiting remarkable potential for forensic analysis (Fig. 7B).

More intriguingly, electrochemical sensing strategies have been integrated with micromotors for sensing applications. Escarpa and colleagues [99] proposed Mg/Au Janus micromotors for detecting targets, which were based on the electrochemical reaction between micromotors and targets (Fig. 7D). More explicitly, when exposed to the chloride-enriched samples, Mg was oxidized because of pitting corrosion and galvanic processes, and then generated hydrogen bubbles and hydroxyl ions. The hydroxyl ions were able to promote the degradation of non-electroactive targets into electroactive molecules, which could be detected with high sensitivity by the difference pulse voltammetry. It was noteworthy that the fluid transport derived from the motion of micromotors greatly improved the analytical signals, thus lowering the detection limits as well as increasing the sensitivity. More recently, Mayorga-Martinez and Pumera [100] fabricated IrO_2_/Pt-composed micromotors as self-propelled tags for sensing based on the electrochemical reaction, as shown in Fig. 7C. More explicitly, IrO_2_, which could enhance electroactivity for hydrogen evolution reaction, played an important role in converting the detection signal into electric signal. In general, the combination of traditional sensing strategies with micromotors is bound to bring revolutionary changes to the sensing fields.

## Applications of Biosensor Micromotors

Micromotors have exhibited unpredictable potential in a variety of fields since their dynamic movements are able to conquer the deficiency of traditional static system, spontaneously enhancing the mass transfer with actively moving matter phenomena. In particular, evolved with outstanding traits such as autonomous movement and tiny size, micromotors have been proven highly dominant as promising candidates for sensing because they can work efficiently even in samples of ultrasmall volume with negligible sample treatment. As a result, micromotors have received tremendous attention toward integrating them with various sensing strategies for promoting the revolution of sensing in different fields. In this section, we reviewed the recent advances of micromotor-based sensing applications in environment science, food safety, and biomedical fields. Here, we will not describe the specific mechanism too much because most mechanisms have been introduced in detail in the previous 2 sections. Instead, we focused on the application examples and the advantages shown by the micromotors.

### Environmental science

**Fig. 8.**
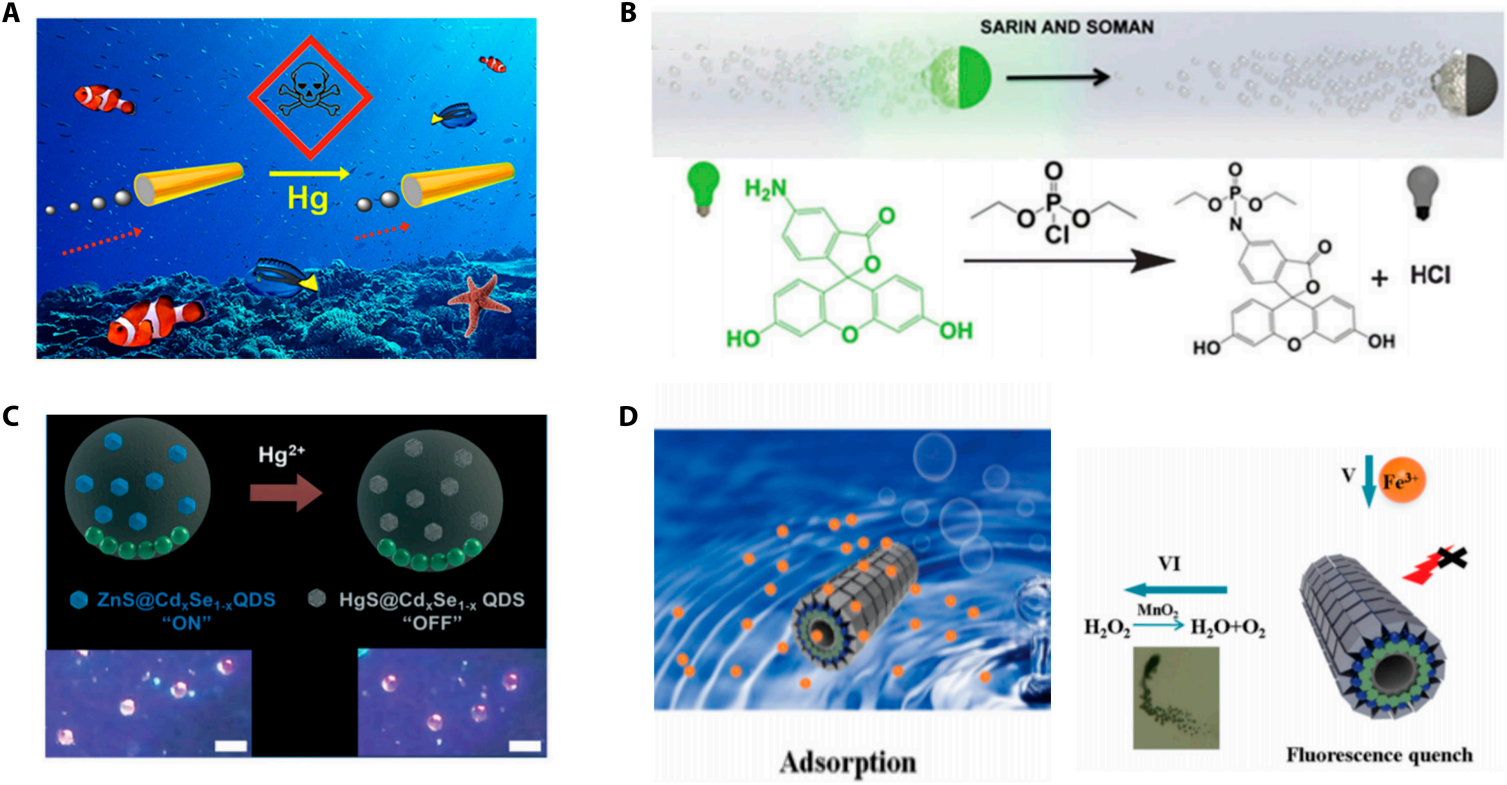
(A) Schematic illustration of microfish for water quality testing. Reprinted with permission from the American Chemistry Society [69]. (B) Mechanism of micromotors for rapid detection of sarin and soman. Reprinted with permission from the Royal Society of Chemistry [87]. (C) Mechanism of “off–on” detection of Hg^2+^ and the images with high resolution captured by the smartphone-based portable device. Scale bars, 50 μm. Reprinted with permission from the Royal Society of Chemistry [79]. (D) MnO_2_-catalyzed bubble-powered micromotor for detecting Fe^3+^ in water. Reprinted with permission from the Royal Society of Chemistry [88].

Nowadays, rapid industrialization has led to excessive discharge of harmful pollutants into water and air, which greatly threats the human health. To alleviate this problem, researchers have been devoting considerable efforts to exploit effective technologies to detect the pollutions, which is prerequisite for the environmental remediation [101,102]. Advance in micromotors has attracted much attention from the field of environmental science by offering outstanding dominance including tiny size and non-invasive and continuous mixing resulting from autonomous movement. These advantages of micromotors allow them to work efficiently in different kinds of samples even of low volume for detecting various pollutants. Up to now, tailoring functional micromotors have been exploited to the environmental science field for sensing toxins, bacteria, heavy metal ions, and so on. As a typical example of micromotors applied in sensing for environmental science, the micromotor called enzyme-powered microfish proposed by Wang and colleagues [69] served as a powerful tool based on speed sensing strategy for water quality testing (Fig. 8A). Similar to the phenomenon that the lifetime and swimming behavior of live fish in nature are dependent on the water quality, the swimming performance and lifetime of these micromotors were affected by toxin in the water. More explicitly, the micromotors were propelled by the bubbles derived from the catalysis of catalase on decomposition of H_2_O_2_. Toxin could lead to the inhibition of the enzyme catalase, which acted as the biocatalytic engine of the micromotor, resulting in obvious time-dependent loss in catalase activity, thus affecting the moving performance. Through observation of the velocity, they demonstrated that the enzyme-powered microfish provided optical sensitive visualization of the variation in the motion performance with the attendance of pollutants involving Hg^2+^, Cu^2+^, sodium azide, and aminotriazole. It was noteworthy that this artificial microfish was capable of addressing ethical issues as well as the reproducibility issues, serving as a powerful tool for real-time testing of water quality. Later, based on the “on–off” fluorescent strategy, they employed bubble-propelled dye-coated micromotors for rapid detection of nerve agents such as sarin and soman simulants, as shown in Fig. 8B [87]. The “on–off” signal variation was triggered by the quenching phenomena of nerve agents on the fluorescent dye. With the autonomous locomotion, the quenching efficiency of nerve agents on the fluorescence was greatly enhanced, making it possible to implement real-time detection. In comparison with the traditional static nerve agent’s detection methods, these micromotors provided a real-time on-site sensing approach, holding great promise for detecting diverse chemical agents. Based on the luminescence quenching mechanism, another bubble-propelled micromotor system with “on–off” luminescence was proposed for efficient sensing of TNT, a highly toxic substance that might cause carcinogenesis and mutagenesis [92]

Based on the fluorescence quenching mechanism of Fe^3+^ on Eu-MOF, Liu and colleagues [88] proposed an “on–off” fluorescent MnO_2_-catalyzed bubble-powered micromotor for detecting Fe^3+^ in water with high sensitivity and excellent selectivity (Fig. 8D). These micromotors were in hollow tubular structure with outer layer of Eu-MOF and inner layer of ethylene diamine tetraacetic acid (EDTA). Notably, benefiting from abundant functional groups, EDTA exhibited strong affinity for heavy metal ions including Fe^3+^. Thus, the micromotors proposed in this work were able to realize the integration of detection and removal of Fe^3+^ in the water sample, serving as a powerful dual-functional tool for sensing and removing of other targets in environmental sciences. In addition, Zuo and coworkers [93] achieved highly sensitive and selective detection of Hg^2+^ through “on–off” fluorescent micromotor sensing system, which was based on the fluorescence quenching triggered by charge transfer from AO to Hg^2+^, providing an ideal platform for detecting harmful metal ions in water.

It is noteworthy that most of the abovementioned fluorescence-based sensing micromotors applied in environmental science must work with high performance optical microscopes that are sophisticated and bulky, which is a main technical drawback and hinders their on-site detection. Aiming at overcoming this problem, a smartphone-based portable device was conceived to assist the micromotors for point-of-care testing of Hg^2+^ in water [79]. This easy-operated platform consisted of a smartphone, emission filters, optical lens, a compartment for lasers insertion, and a movable platform for place of the solution to be detected. With employment of Janus micromotors with fluorescent ZnS@Cd*_x_*Se_1−*x*_ QDs, the “off–on” detection of Hg^2+^ was carried out and images with high resolution were obtained by the smartphone-based portable device (Fig. 8C). It is worth mentioning that similar analytical results were observed through a high-resolution optical microscope, verifying the accuracy of this smartphone-based portable device. In comparison with traditional point-of-care testing (POCT) of Hg^2+^, this strategy enabled detection in just microliters of the sample benefiting from the dominance of micromotors, showing promising potential in superseding the complex microchips or paper-based strips. Intriguingly, this smartphone-based portable device was versatile for different fluorescence-based sensing micromotors to detect diverse targets.

Apart from detection of heavy metal ions and toxins, monitoring of pH of the water is also an important part in environmental science. The micromotor sensor with pH-responsive fluorescent variation provided valuable references for real-time sensing of the pH [38]. Making use of the pH-responsive sensing strategy, Li and colleagues [95] developed a biodegradable FITC-based micromotor for gas sensing. As micromotors only worked in the aqueous environment, only gases such as ammonia (NH_3_) and hydrogen chloride (HCl) that were able to affect the solution pH could be detected by these micromotors. With the autonomous movement, the mixing speed of the solution was accelerated. As the fluorescence intensity of FITC grew with the pH value, the micromotors exhibited strong fluorescence when exposed to the NH_3_ gas environment but exhibited little fluorescence when exposed to the HCl gas environment. More intriguingly, these micromotors mainly consisted of a biodegradable polymer and were powered by the enzyme, which minimized side pollution to the environment. Consequently, micromotor-based sensing platforms usher in a new era of environmental science because the tiny size and autonomous movement of micromotors are able to dilute the shortcomings of the previous sensing strategies.

### Food safety

**Fig. 9.**
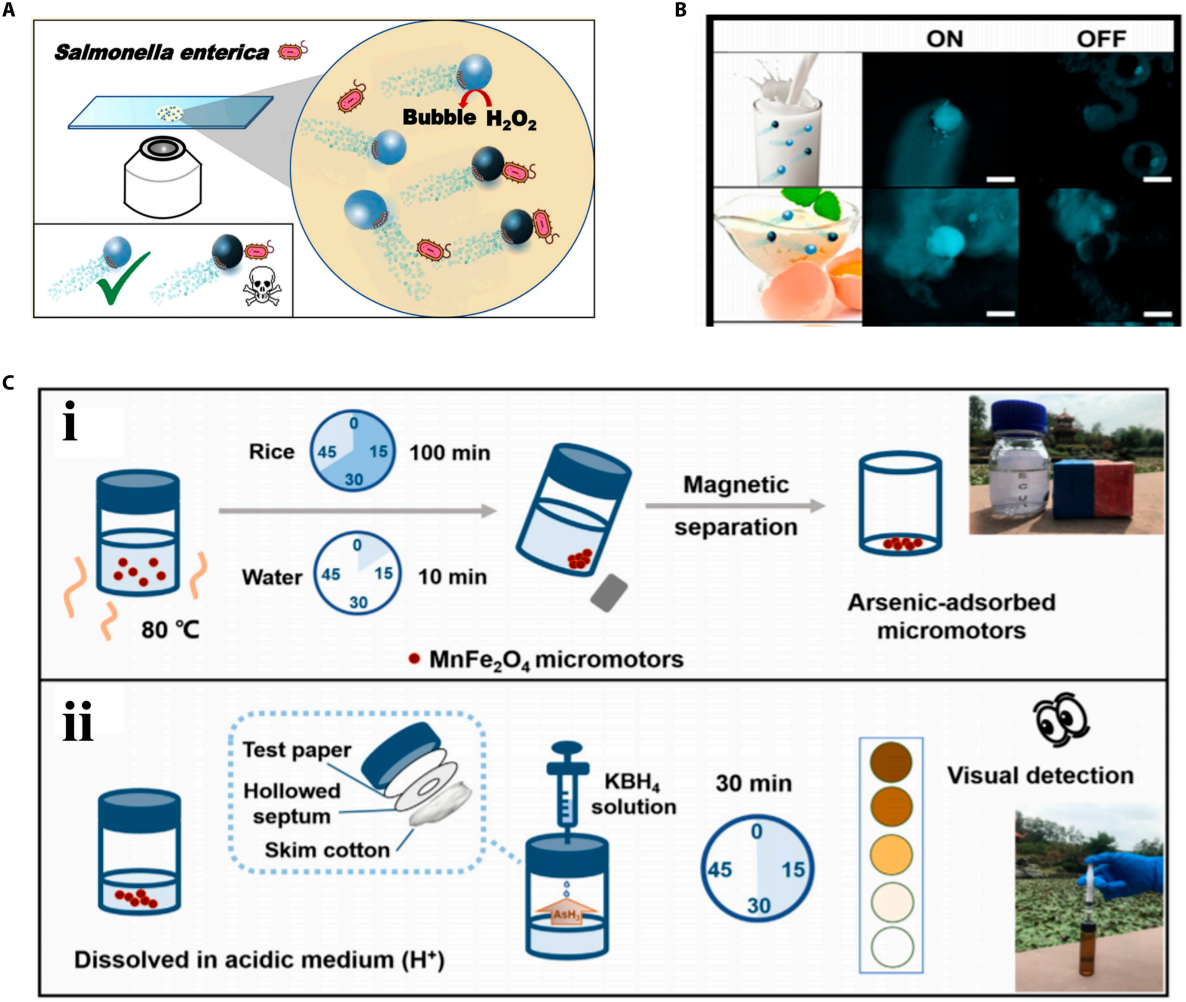
(A) Schematic illustration of QD-composed micromotor system for detecting LPS in food sample. Reprinted with permission from the American Chemistry Society [90]. (B) Detection results in food sample including milk and eggs. Scale bars, 20 μm. Reprinted with permission from the American Chemistry Society [90]. (C) Schematic illustrations of MnFe_2_O_4_ micromotor combined with the Gutzeit method for digestion and detection of arsenic in rice sample. Reprinted with permission from Elsevier [62].

Food safety has been one of the most significant worldwide concerning issues [103,104]. During the last decade, researchers have been trying to employ micromotors to help the relief of the food safety issues. For instance, Escarpa and coworkers [99] utilized a bubble-propelled Mg/Au Janus micromotor to detect and remove the diphenyl phthalate (DPP), which is a common organic pollutant in food samples. As the non-electroactive DPP could be degraded into electroactive phenol, quantification of DPP could be realized by the difference pulse voltammetry. Benefiting from the autonomous movement, the detection was carried out by simply dropping samples into the solution containing navigating Mg/Au micromotors. Furthermore, the autonomous locomotion allowed these micromotors to work efficiently even in viscous samples. They have verified that these micromotors could detect DPP in milk, water, and whiskey samples in short time and with good reproducibility. Apart from organic pollutants, bacteria are also major causes of food safety issues. Effective detection of food samples is an important approach to protect humans from poisoning and infection. Aiming at effective early detection of bacteria in food, Escarpa and colleagues [105] presented GO-composed micromotors for high-performance monitoring of mycotoxins involving ocratoxin A and fumonisin B1 simultaneously. In this work, the 2 specific aptamers of ocratoxin A and fumonisin B1 were decorated with different fluorescent dyes and exhibited quenched state when anchored to the surface of GO. Benefiting from the autonomous movement and specific recognition, the corresponding aptamers were released from the micromotors and the fluorescence was recovered with high efficiency in the presence of target mycotoxins. Compared with the traditional aptamer-based sensors applied in food testing, this micromotor-based sensing system possessed dominances including lower sample volume requirement, less operation, and higher sensitivity, thus resulting in reliable large-scale analysis. Later, they proposed an “on–off” QD-composed micromotor system for detecting lipopolysaccharides (LPS), a normal endotoxin from *Salmonella enterica* (Fig. 9A) [90]. In detail, the 3-deoxy-d-manno-oct-2-ulosonic acid in LPS could bind with the receptor-functionalized QDs, leading to a quick concentration-dependent quenching of the fluorescence on the micromotors. Taking advantage of the spontaneous motion of micromotors, they demonstrated that this platform enabled quick detection of enterobacterial contamination in the raw and viscous food samples by detecting endotoxins, as shown in Fig. 9B. It is noteworthy that this platform could avoid the dilemma when the contaminations were suspected but the organism could not be recovered by bacteriological methods, serving as a reliable platform for practical applications. Moreover, they optimized these micromotor-based sensing systems by imparting the micromotors with magnetics for better control and enhancing the mixture [106].

More recently, Hou and coworkers [62] designed a MnFe_2_O_4_ micromotor combining with the Gutzeit method for digestion and detection of arsenic in rice sample. Notably, arsenic and inorganic arsenic compounds are a common class of carcinogens in rice, which are harmful to human health. Digestion of the arsenic from the solid phase is a prerequisite for detection. In this work, the MnFe_2_O_4_ micromotors could move autonomously in the solution composed of H_2_O_2_ due to the intensive O_2_ bubbles derived from decomposition of H_2_O_2_, which accelerated the mixing speed of the sample. •OH from the reaction between Fe^2+^ and H_2_O_2_ could oxidize organics on the micromotor’s surface into small molecules, leading to the successful digestion of the rice sample. Notably, this was the first micromotor employed for digestion. In comparison with classical sample digestion approaches, the micromotor-involved digestion method obviated the need for large instrument and toxic digestive juice. Furthermore, because materials containing nanomagnetic iron showed strong affinity of arsenic, these MnFe_2_O_4_ micromotors could also act as absorbent for adsorbing arsenic from the sample, which benefited the following analysis of arsenic. As depicted in Fig. 9C, after collecting the MnFe_2_O_4_ micromotors, sensitive visual detection of arsenic was realized by the Gutzeit method. Overall, these MnFe_2_O_4_ micromotors served as a powerful tool for detection of arsenic in rice due to their property of solid-phase extraction, which involved digestion and adsorption. Generally speaking, the emerging micromotors have revolutionized the field of food safety detection due to their tiny size and autonomous movement. By integrating traditional methods with the micromotors, novel detection methods with short detection time, high efficiency, low cost, and no sample preparation could be proposed for applications.

### Biomedical fields

**Fig. 10.**
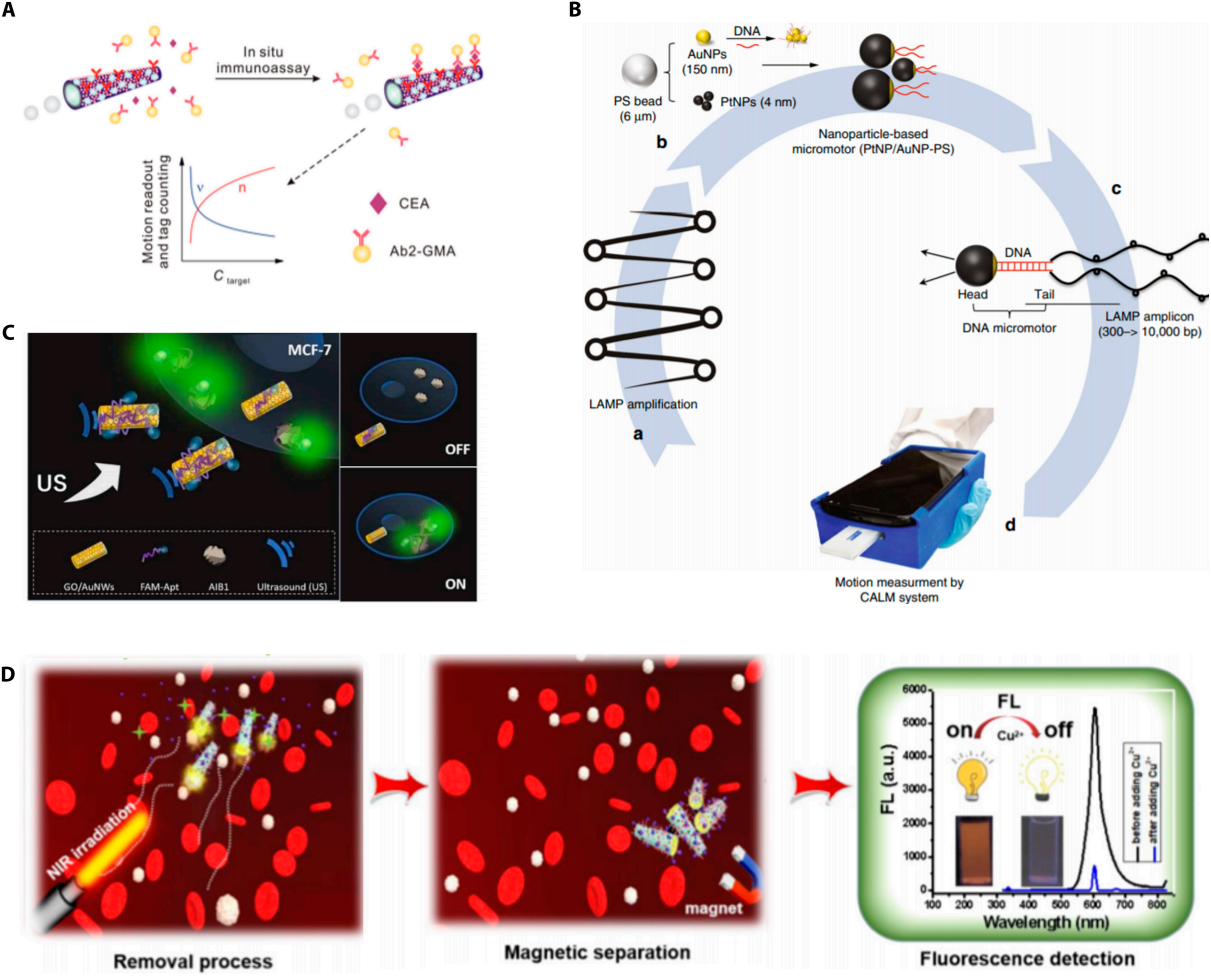
(A) Schematic illustration of double-signal platform for visual in situ immunoassays of tumor. Reprinted with permission from the American Chemistry Society [67]. (B) Cellphone-integrated optical micromotor sensing platform for portable detection of HIV-1. Reprinted with permission from Springer Nature [68]. (C) Schematic illustration of GO-coated micromotors with fluorescence-tagged DNA aptamers for intracellular qualitative determination of AIB1 oncoproteins. Reprinted with permission from John Wiley and Sons [81]. (D) Micromotors for removal/detection of Cu^2+^ in blood. Reprinted with permission from Elsevier [91].

Micromotors have proven highly advantageous as sensors for biomedical fields because their tiny size and autonomous locomotion enable non-invasive detection in biosamples, which are usually of small volume. With enormous scientific efforts, there have been plentiful micromotors proposed for biosensing. It is noteworthy that most of the micromotor-based sensing strategies that are based on the specific recognition of target probes or antigen antibody, which are mentioned in the Stratagems of the sensing micromotors section, can be employed for detection in biomedical areas. To elaborate, biomacromolecules including nucleic acids and proteins could be detected by utilizing specific probes or antibodies, thus greatly benefiting the early diagnosis and prevention of diseases. By way of example, the first micromotor-based sensing platform used for detecting cancer biomarkers was proposed by Yu et al. [67]. This double-signal platform could realize visual in situ immunoassays of tumor markers by counting the tag number and observing speed changes resulting from the selective recognition of antibodies with targets (Fig. 10A). By modifying the surface of micromotors with specific antibodies, tumor markers could be captured, followed by recognition with secondary antibody-modified tags for readout. It is worth mentioning that the large surface of these micromotors allowed for modification of intensive antibodies, which greatly enhanced their biosensing performance. In addition, the double-signal readout ensured the sensitivity and accuracy of the detection, which offered a candidate to practical diagnostic applications. Besides, Wang and colleagues [97] developed a colorimetric micromotor-based immunoassay for detection of cortisol. By immobilizing anti-cortisol on the surface of micromotors, the cortisol was captured by the micromotors and the tags could lead to a colorimetric assay. They demonstrated that the self-driven micromotors could contribute to a more efficient and faster detection process, constructing an efficient and rapid naked eye platform for cortisol detection.

More recently, Escarpa and colleagues [73] designed a micromotor-based fluorescent immunoassay for detecting procalcitonin (PCT). In this work, specific antibodies were bound to the surface of micromotors. With the autonomous movement resulting from bubble propulsion and magnetic guidance, these micromotors could actively recognize PCT and present visual fluorescent signal. They employed these functionalized micromotors for PCT detection in samples from sepsis-suspicious infants. Results indicated that these micromotors were capable of working efficiently in sample of small volume with good feasibility for detecting clinical samples, alluding great potential in point-of-care diagnosis of diseases. Besides, they also described an “off–on” micromotor-based platform for detecting the LPS, an endotoxin related to sepsis. With the competitive affinity of LPS and WS_2_-composed micromotors with rhodamine-labeled peptide, the fluorescence signal went through an “off” to “on” state because of unquenching. This WS_2_-composed micromotor could realize high-selective determination and quantitative recovery of LPS in bacteria cultures and human serum, serving as a compelling tool for real-time detection of clinical-relevant toxins [85]. Based on the competitive affinity, they also tailored micromotors for sensing of cholera toxin B by functionalizing the micromotors with different 2D nanomaterials [107]. As demonstrated, these micromotors were versatile for biosensing because of their feasibility of loading diverse peptides, indicating validity in biomedical areas.

Based on the selective recognition of probes and target sequences, a cellphone-integrated optical micromotor sensing platform was designed for portable detection of human immunodeficiency virus 1 (HIV-1) with high sensitivity. The high sensitivity of this system was ascribed to the introduction of loop-mediated isothermal amplification (LAMP) [68]. To be specific, as depicted in Fig. 10B, nucleic acid of HIV-1 was first amplified through LAMP and then LAMP amplicons were captured by the DNA-modified micromotors by means of selective recognition. As the capture of LAMP amplicons could lead to a decrease in the motion velocity of micromotors, the qualitative assay of HIV-1 could be realized and read out with the help of a cellphone. This micromotor-based sensing platform was sensitive, affordable, and convenient, which acted as a promising tool in early diagnosis and prevention of diseases. In addition, Subjakova and colleagues [81] functionalized the acoustic GO-coated micromotors with fluorescence-tagged DNA aptamers for intracellular qualitative determination of amplified breast cancer 1 (AIB1) oncoproteins (Fig. 10C). These micromotors were driven by the ultrasound to span the cell membrane for efficient intracellular uptake. Due to the competitive recognition of targets with aptamers, the aptamers left the surface of micromotors, leading to a fast fluorescence recovery. In comparison with static methods, these acoustic micromotors enabled more efficient intracellular internalization for AIB1 sensing, which allowed for sensitive early diagnosis of breast cancer.

Furthermore, this competitive aptamer strategy provided valuable reference for novel sensing platforms for early diagnosis of different cancers. Based on the competitive aptamer strategy, micromotor-based sensing platforms also offered candidate to detection of cells. Li and coworkers [94] modified the micromotors with TLS11a aptamers for detecting tumor cells, opening a feasible avenue for real-time and sensitive assays of cells in clinical applications. Also, Escarpa and colleagues [84] proposed an “off–on” micromotor-based sensing platform for determination of *Escherichia coli*. The fluorescent peptides were immobilized on the surface of WS_2_-composed micromotor, exhibiting an “off” state due to the quenching. With the competitive affinity of *Escherichia coli* with the aptamers, the fluorescence was recovered. They verified the feasibility of these micromotors for selective detection in saliva, blood serum, and bacteria media.

Intriguingly, Wan and colleagues [91] recently tailored micromotors for removal/detection of Cu^2+^ in blood, as shown in Fig. 10D. More explicitly, these micromotors were composed of tetraethylene pentaamine (TEPA), gold nanoparticles, magnetic mesoporous silica, heparin, and ZnS·Mn QDs. The TEPA and heparin were loaded in the magnetic mesoporous silica tubes for adsorbing Cu^2+^ and anticoagulation, respectively. These micromotors could move autonomously in the blood because of the self-heating of gold nanoparticles under NIR irradiation. In addition, the ZnS·Mn QDs enabled selective fluorescence monitoring of Cu^2+^. With the synergy of these multiple components, these micromotors could realize efficient selective removal and determination of Cu^2+^ in the blood sample without stirring or ultrasonic method. They proved that these micromotors could realize the removal of Cu^2+^ from blood with a rate of 74.1% and the detection of Cu^2+^ with a detection limit of 0.33 ppm, which indicated that these micromotors could realize the combination of diagnosis and treatment. In general, the emerging micromotors have fostered the development of biomedical areas by offering unique properties involving tiny size and autonomous movement.

## Conclusion and Outlook

In conclusion, micromotors have demonstrated immense promise in sensing fields benefiting from their self-propulsion. The present review was devised to comprehensively review the development of tailoring functional micromotors for sensing. At the beginning, we concisely introduced the propulsion mechanisms, endowing the micromotors with property of autonomous movement. Then, we emphasized on the sensing strategies of micromotors and incorporated them with the micromotors for sensing. After that, we reviewed the applications of micromotor-based sensing platforms in different fields including environmental science, food safety areas, and biomedical areas.

Although much progress has been made in tailoring functional micromotors for sensing, there are still some limitations that create a rift between them and practical applications. First, a majority of the available micromotors realize self-propulsion based on poisonous materials and corrosive chemical fuels, which might contaminate the samples to be detected. Despite the fact that their tiny size enables detection in samples of low volume, tiny size only allows small amount of fuel or catalyst loading, which leads to unsatisfactory motion performance and short lifetime. Besides, tiny size also indicates that micromotors have small surface area for immobilizing functional molecules, thus indirectly affecting the detection sensitivity. Furthermore, most of the present micromotor-based sensing platforms are not flexible and versatile because each platform can only detect one kind of target. Thus, more scientific efforts are needed to clear a path toward employing micromotor-based sensing platforms in practical applications.

To overcome the shortcomings mentioned above, the following perspectives might provide valuable references for future development of tailoring functional micromotors for sensing. First, components including fuel parts of the micromotors had to be biocompatible, nontoxic, and harmless in order to be more compatible for practical applications. Notably, biodegradable materials can obviate the need for subsequent micromotor recycling; thus, they are regarded as a potential candidate for the fabrication of micromotors. To overcome problems resulting from small effective area, more scientific efforts should be paid on the structure design of the micromotors toward large surface–volume ratio. Furthermore, insight regarding micromotors with higher ECE may provide an opportunity to meet the requirement of practical applications because higher ECE results in higher locomotion velocity and enables efficient detection even in sample of large viscosity. In addition, functional materials, such as multifunctional probes and magnetic materials, can be integrated into micromotors to provide possible options for versatile detection of different targets with better control. Also, we should keep on developing novel sensing strategies and integrating them with micromotors, which might bring new hope to different fields. The improvement of instrument for receiving signal and readout can also exert a profound impact on the development of micromotor-based sensing platforms. Especially, integrating micromotor-based sensing platforms with the advanced intelligent devices and Internet provides desirable changes for abating the dependency of sensing on professional institutions, which makes fast on-site detection and POCT possible. Last but not least, we should also focus on the development of material science, environmental science, and other fields, which can in turn advance the development of tailoring functional micromotors for sensing.
